# Maintaining relevance: an evaluation of health message sponsorship at Australian community sport and arts events

**DOI:** 10.1186/1471-2458-14-1242

**Published:** 2014-12-04

**Authors:** Michael Rosenberg, Renee Ferguson

**Affiliations:** Health Promotion Evaluation Unit, School of Sport Science, Exercise and Health, University of Western Australia, (M408) 35 Stirling Highway, Crawley, 6009 Perth, Western Australia Australia

**Keywords:** Sponsorship, Settings, Health messages

## Abstract

**Background:**

Health message sponsorship at community sport and arts events is an established component of a health promotion settings approach. Recent increases in commercial sponsorship of sport and community events has swelled competition for consumer attention and potentially reduced the impact of health message sponsorship. The purpose of this study was to evaluate awareness, understandings and behavioural intentions of health messages promoted at sponsored community sport and arts events.

**Methods:**

Interview and self-administered surveys were completed by 2259 adults attending one of 29 sport and arts events held in Western Australia between 2008 and 2013. The surveys measured participant awareness of the health message promoted at the event, as well as comprehension, acceptance and behavioural intention as a result of exposure to health messages.

**Results:**

Awareness of the sponsored health message was 58% across all sponsored events, with high levels of comprehension (74%) and acceptance (92%) among those aware of the health message. Forming behavioural intentions was significantly related to the type of sponsored message promoted at the event, being female and over 40 years of age. Messages about sun protection and promoting mental health were the most likely to result in behavioural intention.

**Conclusions:**

Health message sponsorship, at least within a comprehensive sponsorship program, appears to remain an effective health promotion strategy for generating awareness and behavioural intention among people attending sport and arts events. Remaining relevant within a modern sponsorship environment appears closely aligned to selecting health messages that promote behavioural action relevant to the sponsored event that are also supported by broader health promotion campaigns.

## Background

Community sport and arts settings offer considerable potential as places to promote a wide range of health-related behaviours through sponsorship [1–4]. From a health promotion settings approach, sponsorship can contribute to building health message awareness and support that may motivate health behaviour and create a receptive climate for healthy public policy [1, 5, 6]. Sponsorship of sport and arts events has also become a popular tool for commercial marketers [7–9], seen by a rise in global commercial sponsorship expenditure from approximately $25 billion in 2001 to over $50 billion dollars in 2012 [10]. Sponsorship expenditure in Australia was estimated to reach about $1 billion [10] in 2013, a relatively small proportion of the global sponsorship market. This increased expenditure by commercial sponsors appears to reflect the benefits they receive from their association with sports and the arts [7, 8, 11, 12], while government sponsorship has largely been used to replace tobacco advertising [1, 6].

Greater commercial sponsorship spending is, in part, related to more governments around the world legislating restrictions on the advertising of tobacco associated with sport to minimise the negative effect on the community [6, 13–16], particularly on children [6]. As a result of tobacco advertising restrictions, opportunities were created for other corporations to shift their marketing budgets into the sponsorship space to build attention, support and loyalty for their brands [3, 17]. Internationally, the finance and telecommunication sectors dominate the sponsorship landscape, although the profile of Australian sponsorship is over-represented by car (13.2%), alcohol (7.4%), government (6.1%) and soft drinks (6%) industries [18]. This increasing investment reflects the ability of sponsorship activities to achieve a range of marketing objectives, the most important of which are increased brand awareness, enhanced brand equity, and sales growth [19, 20].

With increasing overall expenditure, the sponsorship space has become cluttered and more sophisticated tactics have been developed to cut through to target markets, such as venue naming rights, online social media, digital rights and real time engagement with sponsored brand products [7, 14, 21, 22]. Strategies for promoting health messages through sponsorship have evolved along with commercially driven sponsorships [2, 6, 23–26], although with an increasingly smaller fraction of overall sponsorship expenditure. There is, however, little recent evidence available to suggest that health message sponsorship can still compete for audience attention within the modern sponsorship environment [27].

Traditional definitions of sponsorship are built around the exchange of cash or kind to gain access to the commercial potential associated with a venue, event, team or person [28]. More modern applications of sponsorship suggest it is an extremely versatile platform, which enables communication to and connection with a wide range of stakeholder groups and in the process achieves a variety of corporate brand objectives [29, 30]. Sponsorship is different to advertising with sponsorship focused more on alignment with the activities in which individuals are involved, allowing them to naturally develop positive attitudes towards the sponsor [31]. Recently, commercial sponsors have focused on creating brand alignment and relevance through association with sponsored events using technological advances to create new opportunities for people to interact at increasingly more personal levels with their brands while engaged in sponsored events [7, 22]. Modern commercial sponsorship is subtle and indirect, aimed to making consumers more receptive to brand persuasion [31, 32].

Unlike commercial sponsors, health sponsors typically promote a lifestyle message relying on conscious processing, comprehension and cognitive elaboration to be effective [3]. Health message sponsorship is thought to work by either sheer exposure leading to familiarity and positive feelings about a health message, a positive affect transfer from the event to the sponsor; and an increased salience of a belief [3, 33]. Underpinning much of the evidence for health message sponsorship [1, 3, 13] is the hierarchy of effects model, whereby exposure to the health message at a sponsored event, has been shown among audience members to result in increases in awareness, comprehension, acceptance, intention and ultimately behavioural action [33–35]. The literature around the evidence of health message sponsorship effect is limited and within the current sponsorship environment there are few examples of systematic sponsorship of sport and arts events promoting healthy lifestyle messages [27].

Since 1992, The Western Australian Health Promotion Foundation (Healthway) has been offering health sponsorship to sport and arts groups in exchange for opportunities to promote a health promotion message (for example, “Be Active”, “Smarter than Smoking”, “Alcohol. Think Again”) and the provision of healthy environments (for example, non-smoking venues; safe alcohol serving practices and healthy food options) [36]. Healthway attempts to match specific health messages to sponsored activities based on the setting or the audience demographics (for example, outdoor events with sun protection messages; contemporary music events with alcohol prevention messages; surfing events with anti-drug messages) and uses combinations of naming rights, signage, branding, merchandise, announcements, social media, web based promotions and player endorsements to promote health messages [4]. Healthway promotes a range of different health messages at sport and arts events in areas including tobacco control, nutrition, physical activity, sun protection, safe alcohol consumption and mental health. Only one health message is assigned to each event or sponsorship project. Between 2008 and 2012, Healthway awarded 4848 sport (72%) and arts (28%) sponsorships valued at approximately $60 million dollars. These sponsorships ranged from small one-off regional community events to large multi-year sponsorships of state based sporting teams or associations.

Between 2008 and 2013, almost all the health messages promoted through Healthway sponsored events formed a component of a broader health promotion campaign. The “Find 30 every day” physical activity message [37] and the “Go for 2 & 5” [38] nutrition message were part of media campaigns funded by the Western Australian Department of Health between 2002 and 2012. The “SunSmart” message was complimented by media campaigns delivered between 1980 and 2013 by the Cancer Council WA aimed at reducing sun exposure among Western Australian adults [39]. The “Drug Aware” [40], and “Alcohol. Think Again” [41] health messages were part of the Western Australian Drug and Alcohol Authority’s health promotion campaign between 2008 and 2012 that included radio, print and television advertising. The “Quit” campaign has been promoting smoking cessation since the mid-1990s though television, radio and print media [42]. The “Smarter than Smoking” youth based tobacco control campaign has delivered a comprehensive anti-tobacco program that includes television, radio, print and digital media since 1996 [43]. The “Act-Belong-Commit” mental health message is part of a broader Mentally Healthy campaign run by Curtin University that included television as part of its media campaign between 2005 and 2012 [44].

Much of the evidence around the effectiveness of health message sponsorship as a health promotion strategy is drawn from the early work on Healthway’s sponsorship program [1, 3, 13, 17, 36, 45]. The results showed that sponsorship impacted positively on spectator/audience awareness and behavioural intention towards sponsored health promotion messages [3, 13, 36], particularly when accompanied by supporting environmental initiatives [13, 45–47]. However, at the time health messages were not closely aligned with broader health promotion campaigns that included a range of marketing activities, such as television, radio and print media advertising. There was also very limited digital marketing conducted when the earlier data was collected. As commercial sponsorship has continued to increase expenditure through involvement in a wide range of community events, the health message sponsorship opportunity in Western Australia has evolved and remains unique. However, new evidence is required on whether health sponsorship remains effective within a broader health promotion program. The aim, therefore, of this study was to measure the communication effect of Healthway’s sponsorship program among adults attending sponsored sport or arts events between 2008 and 2013.

## Methods

### Study design

The sponsorship program’s impact was measured via a cross-sectional intercept survey that assessed cognitive impact of sponsored health messages among adult attendees at sponsored sport or arts events. The surveys were conducted over two 12 month periods (45%, April 2008-March 2009 and 55%, April 2012-March 2013) from a sample of 29 randomly selected ‘eligible’ sport or arts sponsorship projects. Event eligibility was based upon a sponsorship value of at least $25,000 in a calendar year, as well as the provision of financial support for the promotion of the health message [48, 49]. Depending on the actual level of funding and anticipated attendance at the event, surveys were conducted with audience samples of between 50 and 100 patrons.

At each event, the sponsored health message covered one of seven health areas. With the exception of mental health messages (6%), similar proportions of surveys were collected across the seven different health areas (smoking 19%, nutrition 18%, exercise 14%, sun protection 15%, alcohol 13% and drugs 14%) between 2008 and 2013. The health messages promoted to adults during this time period included “Quit”, “Smoke Free WA”, “Smarter than Smoking”, “Go for 2&5”, “Be Active”, “Find 30 every day”, “SunSmart”, “Alcohol. Think Again”, “Drug Aware” and “Act-Belong-Commit”.

Data collection was conducted by trained field officers who were instructed to randomly approach potential respondents at a time that would not interfere with their spectating/enjoyment of the event. Field officers were placed at strategic locations at each event to ensure they captured the widest audience representation as possible. Selection of respondents for the interview-administered surveys occurred through approaching every x^th^ patron (x was a random number for each event) and inviting the person on the interviewers left or right, alternatively, to participate in the survey. Field officers distributed self-administered surveys during breaks in play, or intermission to patrons within an allocated area. Where practical field officers attempted to approach as many different groups of people as possible. Screening questions included: “Can I just check that you a resident of WA?” followed by “Do you or does anyone in your household have any special or formal connection with the organisers of this event?” and finally “How long have you been at the event today?” In all cases, interviews were discontinued with non-residents, those who had an affiliation with the event, and those who had been at the event for less than 30 minutes. Where applicable, field officers may have also checked that the respondent was over 15 years of age. The UWA Human Research Ethics Committee approved the research protocol.

### Instrumentation

Depending on the nature of the event, either an interviewer-administered or self-administered survey was conducted. Factors such as event location, duration, length of intervals, time of day and budget were taken into consideration when deciding the type of survey to be administered.

Both versions of the survey collected information on respondents’ awareness, comprehension, acceptance and behavioural intention in relation to the sponsored health message at the event. In addition, both surveys captured demographic information.

The items included in the instruments have been used, or modified from existing instruments used in measuring health message cognitive impact [4, 50, 51]. The unprompted health message awareness measure: “Do you recall seeing or hearing any health messages or slogans at today’s event” was asked as the first question in both surveys immediately following the screening questions. Respondents who recalled the message, unprompted, in either the self or interview administered survey were categorised as being ‘aware’ of the message.

If the respondent was aware of the message, their comprehension of the message was investigated (“What do you think the message means, what is it actually saying?”), and, if deemed correct or partially correct, their acceptance of the message (“Do you agree, disagree or have no feelings towards the message?”). Respondents who were aware of the message, comprehended the message, and accepted the message were then asked if they had thought about doing something as a result of seeing or hearing the message. These responses were analysed, and those that related to the health message as either a personal adoption, continuation of the behaviour or an attempt to influence others were categorised as relevant intentions.

### Data analysis

Data were analysed using SPSS for Windows Version 21 and STATA Version 13. Descriptive statistics were conducted to quantify the demographic and cognitive impact (awareness, comprehension, acceptance and intention) data. Cognitive impact variables were calculated as a proportion of each preceding level in the hierarchy (i.e. comprehension was calculated among those who were aware of the message; acceptance was calculated among those who were aware of the message and had comprehended the message etc.).

Individual cross-tabulations were conducted to investigate the cognitive impact variables by event type (sport versus arts), age (<40 years versus >40 years), gender (male versus female), health area (smoking, nutrition, exercise, sun protection, alcohol, drugs and mental health) and survey type (interviewer-administered versus self-administered).

To explore the relationship within the cognitive impact hierarchy, serial logistic regression models were generated to analyse each level of the cognitive impact model (Awareness, comprehension, acceptance and intention) for associations between event type, gender and age, after adjustment for the survey type completed at each event. Results for these analyses are presented in Table 1, as OR (95% CI), with significance set at 5%.

**Table 1:** **Overall and demographic comparisons of the cognitive impact of health message sponsorship**

		Awareness (n = 1315) %		Comprehension ^#^ (n = 977) %		Acceptance ^#^ (n = 898) %		Intention ^#^ (n = 365) %
Total (Sample)		58.2		43.2		39.8		16.2
Total (Preceding level)		58.2		74.2		91.9		40.6
	**%** ^**#**^	**OR** ^**a**^ **(95% CI)**	**%** ^**#**^	**OR** ^**a**^ **(95% CI)**	**%** ^**#**^	**OR** ^**a**^ **(95% CI)**	**%** ^**#**^	**OR** ^**a**^ **(95% CI)**
Event								
Arts	50.8	1.0	74.1	1.0	91.8	1.0	43.1	1.0
Sport	60.1	1.6 (0.8 - 3.7)	75.4	0.9 (0.4 - 1.7)	92.6	1.0 (0.5 – 2.0)	29.6	1.9 (0.6 – 5.6)
Gender								
Male	54.0	1.0	71.8	1.0	88.8	1.0	38.8	1.0
Female	**61.8**	1.4 (1.1 - 1.6)*	76.1	1.3 (0.9 - 1.8)	**94.1**	1.9 (1.2 – 3.1)**	41.9	1.2 (0.9 – 1.6)
Age group								
<40 years	57.5	1.0	73.7	1.0	89.9	1.0	34.5	1.0
>40 years	59.0	1.2 (0.9 - 1.5)	74.9	1.0 (0.8 – 1.4)	**94.0**	2.1 (1.0 - 2.9)*	**46.7**	1.7 (1.3 - 2.3)**

OR odds ratio; CI confidence interval; ^#^Percentage of each preceding level in the hierarchy; ^**a**^Adjusted for event, and survey type; *p < 0.05; **p < 0.01.

Note: Bold text indicates statistical significance.

A second series of logistic regression models were generated to explore the relationship between each level of cognitive impact and sponsored health messages. Each of these models was adjusted for age, gender and the event and survey type. Post hoc analysis was conducted to determine significant relationships between all health message types. Results are presented in Table 2, as p > [z] (95% CI) for all permutations of message comparison, with OR (95% CI) presented for significant relationships (p < 0.05).

**Table 2:** **The relationship between each level of cognitive impact and health messages promoted at sponsored events**

	Awareness (n = 1315) %		Comprehension (n = 977) %		Acceptance (n = 898) %		Intention (n = 365) %	
Total	58.2		74.2		91.9		40.6	
	p > [z] (95% CI)	OR (95% CI)	p > [z] (95% CI)	OR (95% CI)	p > [z] (95% CI)	OR (95% CI)	p > [z] (95% CI)	OR (95% CI)
**Smoking**								
Nutrition	0.16 (−0.4 : 2.1)		0.11 (−1.0 : 0.1)		0.68 (−0.7 : 1.1)		**0.04 (−0.2 : 2.4)**	**3.3 (1.0 : 10.6)**
Exercise	0.59 (−1.0 : 1.8)		0.06 (−1.7 : 0.1)		0.26 (−0.9 : 3.3)		0.08 (−0.1 : 1.9)	
Sun protection	0.08 (−0.1 : 2.3)		**0.01 (−1.8 : −0.2)**	**2.8 (0.9 : 5.3)**	0.12 (−0.2 : 2.0)		**0.00 (2.0 : 4.4)**	**17 (5.5 : 52.3)**
Alcohol	**0.03 (−0.1 : 2.3)**	**3.4 (0.9 : 5.7)**	0.15 (−1.0 : 0.2)		0.52 (−0.7 : 1.3)		0.08 (−0.1 : 1.8)	
Drugs	0.43 (−0.7 : 1.6)		**0.00 (−1.6 : −0.8)**	**0.3 (0.2: 0.4)**	0.85 (−0.9 : 1.2)		0.17 (−0.4 : 2.1)	
Mental health	0.84 (−1.3 : 1.6)		0.73 (−2.0 : 1.4)		0.00 (1.1 : 3.0)	**5.4 (2.0 : 15.1)**	**0.00 (0.9 : 3.2)**	**7.6 (4.1 : 23.4)**
**Nutrition**								
Exercise	0.50 (−1.9 : 0.9)		0.44 (−1.3 : 0.2)		0.32 (−0.9 : 3.0)		0.48 (−1.1 : 0.5)	
Sun protection	0.72 (−1.0 : 1.4)		0.16 (−1.0 : 1.9)		0.12 (−0.2 : − 1.6)		**0.00 (0.9 : 3.1)**	**7.4 (2.5 : 21.9)**
Alcohol	0.47 (−0.6 : 1.3)		0.91 (−0.5 : 0.6)		0.72 (−0.8 : 0.9)		0.41 (−1.1 : 0.4)	
Drugs	0.98 (−0.9 : 0.9)		**0.00 (−1.2 : −0.3)**	**0.5 (0.3 : 0.8)**	0.77 (−0.6 : 0.9)		0.58 (−1.4 : 0.8)	
Mental health	0.60 (−1.6 : 0.9)		0.86 (−1.6 : 1.9)		**0.00 (−1.2 : 2.5 )**	**7.8 (2.9 : 20.7)**	0.09 (−0.1: 1.8)	
**Exercise**								
Sun protection	0.34 (−0.7 : 2.1)		0.72 (−1.2 : 0.8)		0.77 (−2.4 : 1.8)		**0.00 (1.4 : 3.2)**	**9.9 (3.8 : 25.9)**
Alcohol	0.22 (−0.1 : 2.0)		0.39 (−0.5 : 1.3)		0.39 (−2.9 : 1.1)		0.90 (−0.5 : 0.4)	
Drugs	0.13 (−0.5 : 2.2)		0.35 (−1.2 : 0.4)		0.30 (−3.2 : 0.9)		0.98 (−0.9 : 0.9)	
Mental health	0.70 (−1.0 : 1.5)		0.58 (−1.3 : 2.3)		0.42 (−1.2 : 2.9)		**0.00 (−0.5 : 1.8)**	**3.1 (1.6 : 6.2)**
**Sun Protection**								
Alcohol	0.82 (−1.0 : 1.3)		0.15 (−0.2 : 1.4)		0.24 (−1.5 : 0.4)		**0.00 (−3.2 : −1.4)**	**0.1 (0.04 : 0.2)**
Drugs	0.65 (−1.2 : 0.8)		0.57 (−0.9 : 0.5)		0.10 (−1.7 : 0.1)		**0.00 (−3.4 : −1.2)**	**0.1 (0.03 : 0.3)**
Mental health	0.42 (−1.9 : 0.8)		0.43 (−1.0 : 2.4)		**0.01 (0.2 : 2.0)**	**3.1 (1.3 : 7.7)**	0.05 (−2.3 : −0.6)	
**Alcohol**								
Drugs	0.39 (−1.2 : 0.5)		**0.00 (−1.3 : −0.3)**	**0.5 (0.3 : 0.8)**	0.59 (−1.1 : 0.6)		0.97 (−0.9 : 0.9)	
Mental Health	0.21 (−1.7 : 0.4)		0.89 (−1.6 : 1.9)		**0.00 (−0.9 : 2.5)**	**5.6 (2.2 : 14.0)**	**0.00 (0.4 : 1.9)**	**3.2 (1.6 : 6.5)**
**Drugs**								
Mental Health	0.55 (−1.4 : 0.7)		0.30 (−0.8 : 2.6)		**0.00 (1.2 : 2.7)**	**7.1 (3.0 : 16.3)**	**0.03 (0.9 : 2.2)**	**3.1 (1.1 : 8.8)**

OR odds ratio; CI confidence interval; Regression models were adjusted for type of event attended, survey, age and gender.

Note: Bold text indicates statistical significance.

To account for recruitment of participants at sport and arts events, all logistic regressions models included a clustering adjustment at the level of event attended. To further increase the robustness of our analysis, we applied bootstrapping (1500 repetitions) to all logistic regression models.

## Results

A total of 2259 people attending a sport (n = 1802; 80%) or arts (n = 457, 20%) event completed either the self (n = 1345; 60%) or interview (n = 914; 40%) administered survey. The sample comprised more females (n = 1221; 54%) than males (n = 1038; 46%), and a similar proportion of people under (1171; 52%) and over (1088; 48%) the age of 40.

Table 1 shows the cognitive impact of promoted health messages across all sponsored events, as a proportion of the sample overall and of each preceding level. Among all respondents, awareness at sponsored events was 58.2%, with females 1.4 times more likely than males to be aware of the sponsorship health message. Comprehension of the health message was high at 74.2% and similar across event type, gender and age. Acceptance of the sponsored health message was also high (91.9%), with females and respondents over 40 years of age significantly more likely to accept the sponsorship health message compared with males and participants younger than 40 years of age respectively.

Forming a relevant behavioural intention following acceptance of the health message was observed among 40.6% of respondents across all sponsorships, with those over the age of 40, 1.7 times more likely to form a relevant behavioural intention compared with people under 40 years of age.

Table 2 shows the cognitive impact hierarchy analysed by the seven different types of health messages promoted at the sponsored events. The level of awareness of the sponsored health message was similar across health messages, after controlling for respondent’s gender, age, survey type and the type of event they attended.

Differences were observed in the proportion of respondents comprehending different types of health messages, with anti-drug messages less likely to be comprehended than smoking, nutrition, and alcohol messages. Sun protection messages were also two and a half times more likely to be understood than anti-smoking messages.

High levels of agreement with the message were found across most of the health messages, with mental health and sun protection messages more likely to be accepted than anti-smoking messages.

The likelihood of forming a behavioural intention towards the message varied greatly with the type of sponsored health message, with sun protection and mental health messages most likely to result in positive behavioural intentions compared with other sponsored health messages. Smoking messages were the least likely to result in a positive behavioural intention compared with other messages except when compared with nutrition and anti-drug health messages.

## Discussion

The results of this study suggest that when implemented as part of a comprehensive sponsorship program, health message promotion at community sport and arts events can reach over half (58%) of audience members and generate behavioural intentions through cognitive processing related to the sponsored health message. These findings are important as health message sponsorship represents a small fraction of the overall sponsorship market, and commercial sponsorship has increasingly been competing for the same audience attention at community events [10, 18, 52]. The results in this study also show that awareness of health messages at sponsored events is similar to those found a decade earlier [3, 13, 49], with the results extending the earlier research by also showing that variations exist in the communication effects of different types of health messages through the cognitive impact hierarchy. However, there is a real possibility that the cognitive impact observed in this study may have already been formed from participants’ exposure to the health message prior to attending the event. Unlike earlier research based upon Healthway’s sponsorship program between 1992 and 2002, the 2008–2013 sponsorship program benefited from broader mass media marketing activity of the health messages promoted at sponsored events through concurrent community wide health promotion campaigns. Experiences in commercial sponsorship show that the combination of advertising and sponsorship is very effective, with sponsorship believed to make consumers more receptive to the influence of the sponsored brands advertising [28, 32]. The finding that sponsored health messages were able to maintain similar levels of cognitive impact over a 15 year period, even with large increases in commercial sponsorship activity, is encouraging, although the findings are impacted by the broader comprehensive health promotion approach, of which sponsorship is one of several strategies.

Regardless of whether awareness was generated through exposure to health messages at sponsored events, or through the wider media activity of health promotion campaigns, the results of this study reinforce previous findings that the sponsored message itself is critical in generating a communication effect beyond awareness. In this study, sun protection and mental health messages resulted in the highest levels of behaviour intention, while smoking and anti-drug messages were the least effective. Health messages promoting healthy nutrition, exercise and alcohol behaviours were moderate and similar in their effectiveness in terms of generating behavioural intention. Differences in the cognitive impact of health messages may be due to message framing [24, 53], however, a modern view of sponsorship would suggest that while messages may independently elicit a strong cognitive impact, spectators will be more receptive to the health message if they perceive a natural fit with the sponsored event activities [11, 21, 54]. Commercial sponsors have recognised this benefit and take a strategic approach to placing their brand alongside other sponsors’ products and associating their brand with a positive event experience. For example, the telecommunication company O_2_ focus on being involved in the enjoyment of the event through social communication based services [7], and beer and food companies negotiate service contracts as part of their sponsorship agreement [52, 55, 56]. How spectators perceived the relevance of the health message at sponsored events was not measured as part of this study, but it is plausible that sun protection and mental health messages were perceived as being well aligned with the sponsored event, while spectators may not have so easily associated messages such as “Quit”, “Smoke Free WA”, “Smarter than Smoking” or “Drug Aware” with sponsored event activities.

In this study, health message sponsorship was proposed to operate through the cognitive impact hierarchy, however, where relevant behavioural intention could be actioned at the sponsored event, high levels of intention were elicited. For example, applying sunscreen (“SunSmart”) as a form of sun protection while attending an outdoor event, or engaging in sport and arts events to be mentally healthy (“Act-Belong-Commit”). Health messages promoting nutrition (“Go for 2 & 5”), exercise (“Find 30 every day”) and alcohol (“Alcohol. Think Again”) behaviours were also able to elicit a moderate cognitive impact response to the level of behavioural intention among spectators which may have been supported by opportunities to be active, the availability of healthy food, and low-strength alcohol options at sponsored events. In contrast, health messages that promoted smoke free environments (“Smoke Free WA”), quitting smoking (“Quit” and “Smarter than Smoking”), or illicit drug education (“Drug Aware”) may have been more difficult to act upon during the event and reflected in lower behavioural intentions. Furthermore, in Western Australia, smoking is prohibited at sponsored events [6] and consuming drugs is illegal. Framing abstinence and cessation of smoking or illicit drug messages that provide opportunities for behavioural action is nonsensical in this environment and already enforced on spectators [24]. The relationship between supportive environments and health behaviours is well known in health promotion [23, 57], and in commercial sponsorship, sport and arts settings are ideal places to create associations between spectator enjoyment and opportunities to engage with the brand in order to provide an advantage over competitors when marketing the product [7, 8, 12, 20, 58]. The extent to which health message sponsorship operates in this way is unknown, although the results of this study offer some insight into the interrelated nature of the sponsored event, framing of the health message, its alignment with event activities, as well as the role of supportive environments to encourage positive health behaviours.

### Limitations

The results of this study should be interpreted with consideration of the following limitations. Survey participants were selected among audience members who had left the event viewing area for a variety of reasons (toilet, food, drink, interval, half time…). There is a chance that differences may exist in the level of awareness of sponsored health messages between spectators who remained engaged in the event and people who moved away from a viewing position. Data were collected among adults using two surveys (self and interviewer-administered) and in some cases sponsorship signage may have been visible to respondents while completing the questions about awareness of health message sponsorship. While interviewers attempted to reduce views to visible signage by positioning the respondent away from signage during the interview, self-administered surveys cannot be controlled in this manner. In addition, awareness may be higher due to prior exposure of the health message. Awareness levels may also have been influenced by the use of only unprompted recall in both the self-administered and interview-administered surveys. It is also possible prior exposure may have influenced respondents’ comprehension, and intention related to the sponsored health message. To minimise the impact of this difference, the type of survey (interview or self-administered) completed was considered in the data analysis. A further limitation of the research design was the cluster sampling design that resulted in a similar health message exposure among people attending the same event. As sponsorship activities were event specific and disproportionate numbers of respondents were drawn across events, cognitive impact may reflect a group rather than individual experience. To reduce the influence of this effect, all analysis was adjusted for the clustering of the event.

## Conclusions

The results suggest that sponsorship at community sport and arts events to promote health messages remains an effective health promotion strategy. However, whether health message sponsorship remains effective without support from a wider health promotion campaign including activities such as media advertising is unclear. Since the original findings were published on the effectiveness of Healthway’s sponsorship program, the commercial sponsorship market has changed dramatically, with considerably greater investment and competition for space with health message sponsorship. This study also considered that message relevance and promotion at sponsored events is possibly now more important than just focusing on raising health message awareness. Additionally, the interaction between supportive environments and health message engagement may offer new opportunities for greater direct engagement in health behaviours at sponsored events. In using health messages from wider health promotion campaigns, sponsorship should be viewed as part of the marketing mix where messages are aligned to relevant activities and engagement at sponsored events is used to increase community receptiveness to health behaviour change.
